# Seed priming improved salt-stressed sorghum growth by enhancing antioxidative defense

**DOI:** 10.1371/journal.pone.0263036

**Published:** 2022-02-25

**Authors:** Xiaoqian Guo, Wenfang Zhi, Yuntong Feng, Guisheng Zhou, Guanglong Zhu

**Affiliations:** 1 Joint International Laboratory of Agriculture and Agri-Product Safety, Yangzhou University, Yangzhou, Jiangsu Province, China; 2 Jiangsu Provincial Key Laboratory of Crop Genetics and Physiology, Yangzhou University, Yangzhou, Jiangsu Province, China; Huazhong Agriculture University, CHINA

## Abstract

Seed priming is regarded as a beneficial and effective method enhancing performance of plants grown under stress conditions. This study illustrated the effect of four seed priming agents (2% H_2_O_2_, 52 mM NaCl, 50 mM KCl, 250 mM MgSO_4_) on two sorghum cultivars (Canada sorghum CFSH-30 and sorghum ‘1230’) grown in saline soils. Sorghum growth characteristics and biochemical parameters were investigated. Seed priming treatments alleviated the adverse effects of salt stress by decreasing MDA content and enhancing antioxidant enzymes (CAT, POD and SOD) activities and proline content, and hence increased sorghum fresh and dry weight. In terms of various parameters, sorghum ‘1230’ was more suitable to be grown in saline soil, and 52 mM NaCl and 50 mM KCl were the optimum priming agents to improve the performance of salt-stressed sorghum.

## Introduction

Salinity is the one of the main abiotic factors restricting plant growth, development and productivity. More than 6% of the world’s lands are saline and ~20% of irrigated lands are currently affected by salt [1, 2]. Human activities and geological processes mainly lead to salinity problem [3]. The various factors that cause salinity stress include poor cultivation practices, irrigation with saline water, less rain fall, high surface evaporation, and weathering of parent rock materials [4]. It is estimated that by 2050, more than 50% of the cultivable lands will be affected by rising soil salinization [5]. Soil salinization is a noticeable environmental problem all over the world. Rational and efficient utilization of marginal lands has become a global issue of concern [6].

Planting salt-tolerant crops in saline soils is one of the feasible ways to maximize the utilization and restoration of saline soils [7]. The selection of salt-tolerant plants and the investigation into salt tolerance mechanisms have become heated research topics for plant breeders and physiologists [8]. Sorghum is the fifth most important cereal crop used as human food, animal forage, and biofuel production [6]. Sorghum is a moderately salt-tolerant and drought-tolerant crop, and there are genotypic differences in response to these two stresses [9]. Sorghum can be planted on compact soil within a wide range of salinity [10] and pH (5.0–8.5) values [11]. Given the economic value and potential tolerance of sorghum, it has a great potential as a food source in areas affected by drought and/or salinity stress.

Seed priming is a long-lasting and effective technique that can improve the performance of plants under abiotic stresses, in terms of physiological and biochemical changes. Seed priming is generally defined as the controlled hydration of seeds, which allows pre-germinative metabolic activities while avoiding the surfacing of the radicle [12]. Seed priming comprises of hormonal priming, osmopriming, nutrient priming, hydropriming, and redox priming [13]. Reports showed that under salinity conditions, seed priming with H_2_O_2_ had better germination, higher photosynthetic efficiency and proline content and reduced oxidative damages caused by ROS [14]. NaCl-priming enhanced soluble carbohydrate and proline content, antioxidant enzyme activity and reduced the damage of muskmelon seed membranes [15]. The application of KCl and KOH through seed priming significantly improved the germination percentage, seedling shoot length and root length, and seedling fresh and dry biomass of *Pisum sativum* [16]. Compared with other priming agents produced by salt solutions, striga seeds conditioned with MgSO_4_ maintained the highest germination regardless of their concentrations [17].

The effects of priming agents varied in terms of various priming agents, concentrations of priming agents, and plant varieties. Suitable priming agents at appropriate concentrations are the key factors to seed priming. Therefore, the current study was conducted to evaluate the responses of sorghum varieties to four seed priming agents planted in saline soils, and to screen out appropriate tolerant varieties for salt-affected regions and the suitable priming agent on the basis of physiological and biochemical parameters.

## Materials and methods

### Experimental design

A field experiment was conducted in Dafeng Coastal Forest Farm, Dafeng County (33°20′N, 120°47′E), Yancheng City, Jiangsu Province, China in 2018. Before starting the experiment, soil samples from a depth of 20 cm at the site were collected to determine basic properties. The soil is classified as loamy soil with a textural class of clay loam and contains 19.75 g kg^-1^ organic matter, 0.72 g kg^-1^ total nitrogen, 1.45 mg kg^-1^ available phosphorus, 279 mg kg^-1^available potassium, and 1.68 g kg^-1^ soluble salt, with a pH reading of 8.8.

The experimental variables were two sorghum varieties, including Canada sorghum CFSH-30 (V1) and sorghum ‘1230’ (V2), and four seed priming agents including 2% H_2_O_2_ (P1), 52 mM NaCl (P2), 50 mM KCl (P3), 250 mM MgSO_4_ (P4). The unprimed seeds served as the control (C). The study was arranged in a 2-factorial completely randomized block design with three replications. There were 30 plots in total. The area of each plot was 30 m^2^ (15 m × 2 m). Sorghum seeds were directly sown in the field at the seeding rate of 20 kg ha^-1^. A total of 225 kg N ha^-1^ as urea and 120 kg ha^-1^ as P_2_O_5_ were applied, with 50% at sowing and 50% at the seedling stage. In the case of seed priming, sorghum seeds of two varieties were soaked for 48 h at 15°C in each of the treated solutions, immediately followed by drying seeds back at room temperature to the original seed moisture content. Other field practices including in the control of insects, diseases and weeds were in conformity with local recommendations.

### Plant sampling and analysis

Five plants of each plot were collected at elongation, flag leaf, booting, and heading stages, respectively. After measuring fresh weight, all the samples were dried in the oven at 105°C for 30 min to deactivate enzymes and then at 80°C to constant weight to measure dry weight.

Another five plants of each plot were sampled for leaves to determine the activity of catalase (CAT), peroxidase (POD) and superoxide dismutase (SOD) and the content of proline and MDA. These samples were soaked in liquid nitrogen first and then stored in a low-temperature freezer (-80°C). The activity of CAT, POD, and SOD was measured according to the method of Bergmeyer [18], Upadhyaya et al. [19], and Beauchamp and Fridovich [20], respectively. The content of proline was measured using the method of Bates et al. [21]. The content of MDA was determined according to Zhang et al. [22].

### Statistical analysis

The experimental data were calculated with Excel 2016 and graphed with SigmaPlot 10. Statistix 9 was used to conduct the analysis variance (ANOVA) in a completely randomized design in a factorial arrangement (genotypes × seed priming). The LSD test was used to determine the comparison of means at the 5% probability level.

## Results

It can be seen from Table 1 that variety, seed priming, and their interaction produced significant effects on fresh weight during the whole growth period, except that the interaction of variety and seed priming had no significant effect on fresh weight at heading stage. For V1, P3 had a better effect on promoting fresh weight at elongation, flag leaf, booting, and heading stages, and the fresh weight reached 84.1, 194.4, 294.6, 272.0 and 201.6 g plant^-1^ respectively. As for V2, the fresh weight of V2P3 was the highest at elongation and heading stages, reaching 112.9 and 385.0 g plant^-1^, respectively. At flag leaf and booting stages, however, P2 produced the highest fresh weight, reaching 228.9, 389.6 and 314.5 g plant^-1^, separately. During the whole growth period, fresh weight experienced a significant increase before booting stage, and declined after heading stage.

**Table 1:** Effect of seed priming on fresh weight of sorghum under salt stress at different growth stages.

Variety	Seed priming	Fresh weight (g plant^-1^)			
		Elongation	Flag leaf	Booting	Heading
**V1**	**C**	64.5 e	136.7 cd	212.1 de	228.6 ef
	**P1**	62.1 e	185.6 b	181.8 e	221.3 ef
	**P2**	63.9 e	132.2 d	190.9 e	243.6 de
	**P3**	84.1 d	194.4 b	294.6 c	272.0 cd
	**P4**	80.7 d	138.9 cd	201.0 de	196.6 f
**V2**	**C**	90.9 cd	131.1 d	238.5 d	338.3 b
	**P1**	105.4 ab	205.6 b	369.5 ab	372.8 a
	**P2**	90.6 cd	228.9 a	389.6 a	363.0 ab
	**P3**	112.9 a	158.9 c	365.1 ab	385.0 a
	**P4**	98.4 bc	188.9 b	323.0 bc	292.4 c
**Variety**		**	**	**	**
**Seed priming**		**	**	**	**
**Variety×Seed priming**		*	**	**	ns

Note: Different lowercase letters in the same column indicate statistical difference at the 0.05 probability level. V1: CFSH30, V2: 1230, C: control, P1: H_2_O_2_, P2: NaCl, P3: KCl, P4: MgSO_4_, ns: not significant

*: statistical difference at the 0.05 probability level, and

**: statistical difference at the 0.01 probability level. The same as below.

The results in Table 2 showed that variety had significant effects on dry weight at elongation, flag leaf, and heading stages. The effect of seed priming on dry weight reached a significant level in all the growth periods. Their interaction affected sorghum dry weight significantly at elongation, flag leaf and booting stages. In comparison to V1, the dry weight of V2 was relatively high. For V1, compared to control and other seed priming agents, P3 increased dry weight by 27.2%, 30.5%, and 32.3%, respectively at the stages of booting, and heading. Also for V2, P3 remarkably increased dry weight at most growth stages, with an increase of 12.7% and 35.6% at the stages of elongation and heading. P1 and P4 decreased dry weight of V2 while remarkably improved the dry weight of V2. Among all the treatments, V2P3 had the highest dry weight (155.2 g plant^-1^), followed by V2P1 and V2P2 (141.0 and 137.9 g plant^-1^), respectively.

**Table 2:** Effects of seed priming on dry weight of sorghum under salt stress at different growth stages.

Variety	Seed priming	Dry weight (g plant^-1^)			
		Elongation	Flag leaf	Booting	Heading
**V1**	**C**	13.3 cd	28.2 bcd	75.5 bcde	99.9 def
	**P1**	12.5 de	37.5 a	64.6 de	93.8 ef
	**P2**	13.0 cde	31.4 b	68.9 cde	102.2 cde
	**P3**	11.4 e	26.8 cd	96.0 a	130.4 b
	**P4**	14.5 bc	24.9 de	76.4 bcde	85.0 f
**V2**	**C**	15.0 b	21.6 e	61.8 e	113.8 cd
	**P1**	15.2 ab	28.3 bcd	84.7 abc	133.2 b
	**P2**	12.7 de	30.3 bc	85.3 ab	132.2 b
	**P3**	16.9 a	25.4 de	78.2 bcd	154.3 a
	**P4**	15.8 ab	30.4 bc	74.8 bcde	118.0 bc
**Variety**		**	*	ns	**
**Seed priming**		*	**	*	**
**Variety×Seed priming**		**	**	**	ns

**Fig 1:**
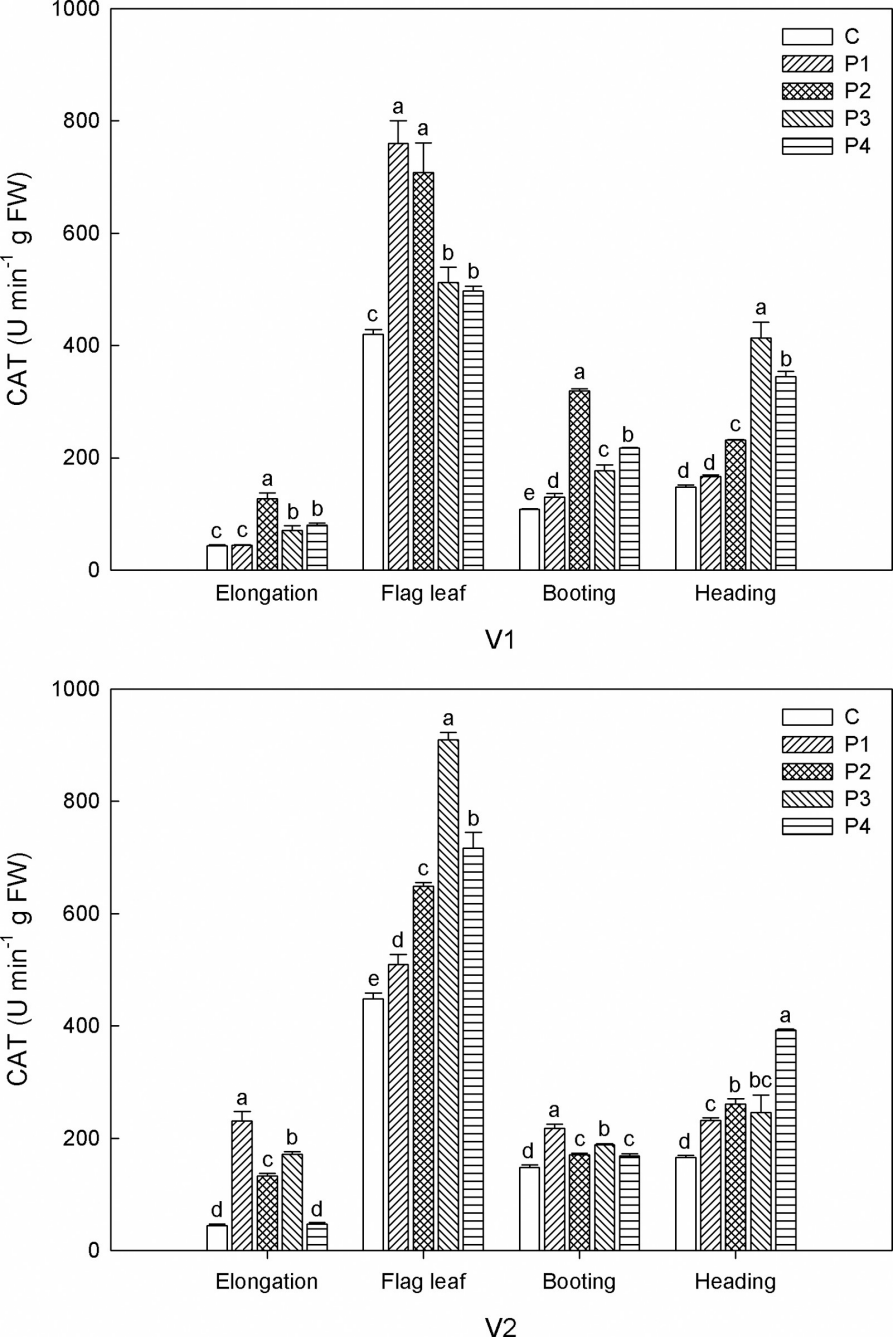
Effects of seed priming on CAT under salt stress at different growth stages. V1: CFSH30, V2: 1230, C: control, P1: H_2_O_2_, P2: NaCl, P3: KCl, P4: MgSO_4_. Within the same growth stage, the bars marked with different lowercase letters are statistically different at the 0.05 probability level.

All the seed priming treatments enhanced CAT activity to varying degrees (Fig 1). For V1, CAT activity treated with P2 came top with 127.5 U min^-1^·g FW at elongation stage, 707.8 U min^-1^·g FW at flag leaf stage, and 319.4 U min^-1^·g FW at booting stage. This was followed by P3 and P4 at the same stage. At flag leaf stage, CAT activity treated with P1 was a little higher than that treated with P2, but the difference was not significant. At heading stage, P3 showed the greatest promoting effect and increased CAT activity to 413.7 U min^-1^·g FW. As for V2, P1 played a vital role in improving CAT activity at elongation and booting stages. While at flag leaf and heading stages, the highest CAT activity was observed with the treatment of P3 and P4 respectively. Throughout the growth period, CAT activity increased remarkably, reaching its peak at flag leaf stage and then declined gradually.

**Fig 2:**
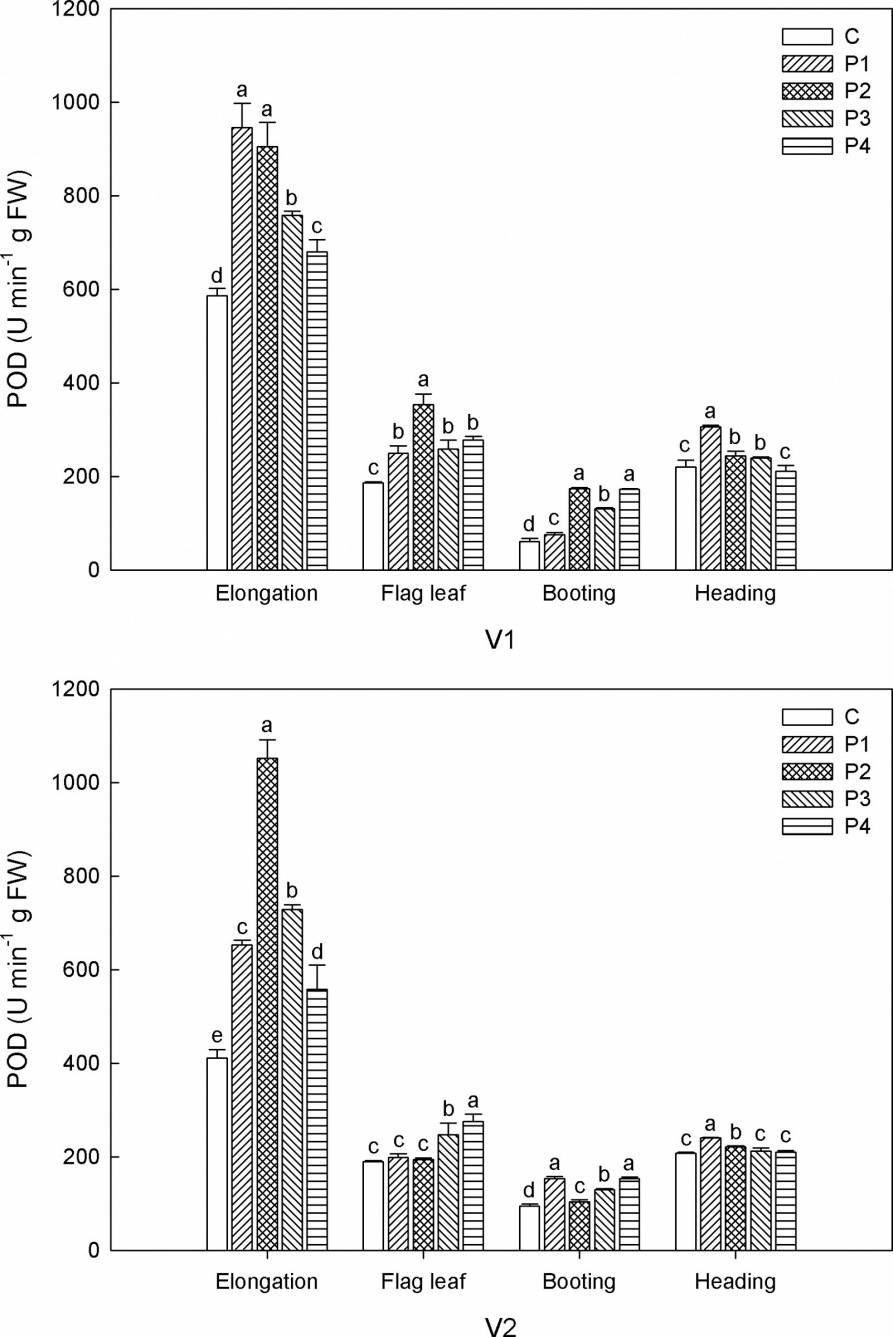
Effects of seed priming on POD under salt stress at different growth stages. V1: CFSH30, V2: 1230, C: control, P1: H_2_O_2_, P2: NaCl, P3: KCl, P4: MgSO_4_. Within the same growth stage, the bars marked with different lowercase letters are statistically different at the 0.05 probability level.

The POD activity of V1 was significantly increased by all seed priming agents except for P4 at heading stage (Fig 2). The largest increase in POD activity was shown in P1 at elongation and heading stages (359.3 and 85.2 U min^-1^·g FW, respectively), and in P2 at flag leaf and booting stages (166.7 and 111.9 U min^-1^·g FW separately). While for V2, P2 had the best effects on increasing POD activity at elongation stage. Compared with the control, P3 and P4 significantly enhanced POD activity (247.5 and 276.3 U min^-1^·g FW respectively) at flag leaf stage whereas P1 and P2 had no significant effects. At heading stage, P1 and P2 increased POD activity significantly (241 and 221.4 U min^-1^·g FW respectively) while P3 and P4 did not have significant effects. Compared with other seed priming agents, P1 and P4 had better promoting effects at booting stage. The activity of POD experienced a sharp decrease from elongation stage to flag leaf stage and was kept relatively stable after flag leaf stage.

**Fig 3:**
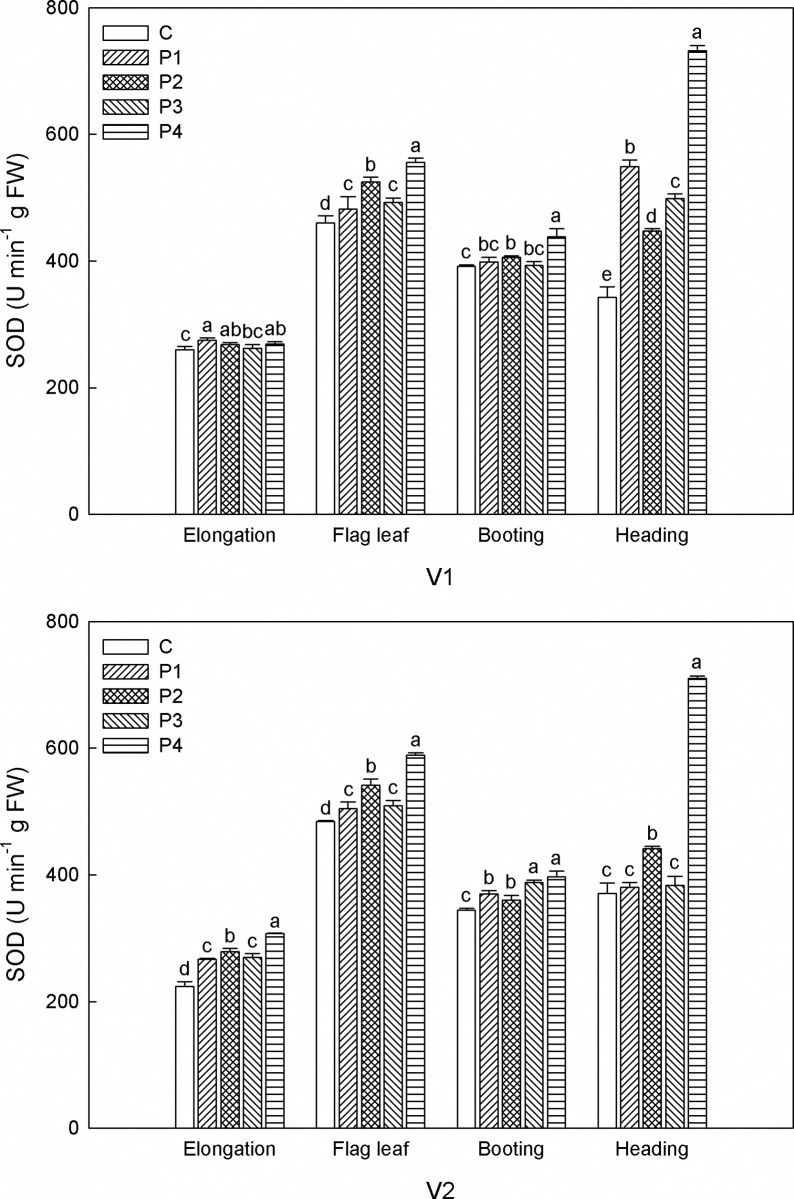
Effects of seed priming on SOD activity of sorghum plants under salt stress at different growth stages. P1: control, P2: H_2_O_2_, P3: NaCl, P4: KCl, P5: MgSO_4_. Within the same growth stage, the bars marked with different lowercase letters are statistically different at the 0.05 probability level.

In comparison with other seed priming treatments (Fig 3), P4 showed the best promoting effects on SOD activity in V1 and V2, reaching 269.1, 556.1, 438.7 and 731.6 U min^-1^·g FW, respectively in V1 and 307.4, 588.7, 397.3 and 710.4 U min^-1^·g FW, separately at V2. At elongation stage, P1, P2, and P4 increased SOD activity in V1, but there was no difference between them. Similar situation was observed in V2 treated with P3 at booting stage. P2 ranked the second in increasing SOD activity, except for V1 at elongation stage and heading stage. At heading stage, SOD activity in V1 treated with P1 ranked the second, reaching 549.0 U min^-1^·g FW. SOD activity generally increased with plant growth though there were fluctuations. Notably, SOD activity in V1 was remarkably increased by P1 and P4 at heading stage, overtaking that at flag leaf stage and finally reaching its peak of 549.0 and 731.6 U min^-1^·g FW, respectively. P4 significantly improved SOD activity of V2 at heading stage to a high level, although SOD activity treated with other seed priming tended to be gentle after booting stage.

The effects of variety, seed priming, and their interaction on proline content were significant (Table 3). Compared to V2, V1 had higher proline content. Seed priming increased proline content of V1 at elongation and heading stages, but there is no significant difference among the four seed priming agents at elongation stage. Proline content of V1 did not increase with P4 treatment at flag leaf stage and with P1 treatment at booting stage. P1 had the largest increase in proline content of V1 at flag leaf stage (87.4 μg g^-1^ FW), followed by P2 and P3 at 85.9 and 77.6 μg g^-1^ FW, respectively. At booting stage, the highest proline content was obtained at P4. P3 increased proline content of V2 to the greatest level at most growth stages (98.7 μg g^-1^ FW at elongation stage, 73.2 μg g^-1^ FW at flag leaf stage and 38.1 μg g^-1^ FW at heading stage). At booting stage, proline content was remarkably increased by P1 while other three seed priming agents had no significant effects. Among all the treatments, the highest proline content was observed in the V1P4 treatment at elongation and booting stages, in the V1P1 treatment at flag leaf stage, and in the V1P3 treatment at heading stage.

**Table 3:** Effects of seed priming on proline of sorghum under salt stress at different growth stages.

Variety	Seed priming	Proline (μg g^-1^ FW)			
		Elongation	Flag leaf	Booting	Heading
**V1**	**C**	74.8 de	75.6 cd	26.6 cd	29.2 d
	**P1**	121.8 a	87.4 a	25.1 de	37.9 b
	**P2**	117.2 a	85.9 ab	29.5 b	30.4 cd
	**P3**	112.7 a	77.6 bc	27.4 c	52.4 a
	**P4**	122.0 a	71.3 cd	32.1 a	37.0 b
**V2**	**C**	72.7 de	44.2 f	20.2 f	31.3 cd
	**P1**	68.8 e	58.7 e	24.6 e	37.8 b
	**P2**	82.4 cd	66.6 de	21.4 f	32.6 c
	**P3**	98.7 b	73.1 cd	22.0 f	38.1 b
	**P4**	92.8 bc	72.0 cd	21.5 f	32.3 c
**Variety**		**	**	**	**
**Seed priming**		**	**	**	**
**Variety×Seed priming**		**	**	**	**

Variety, seed priming, and their interaction significantly affected MDA content at elongation and flag leaf stages (Table 4). At booting and heading stages, only seed priming had significant effects on MDA content. Seed priming reduced MDA content of both V1 and V2. The largest reduction in MDA content of V1 was recorded with at P1 at elongation, booting, and heading stages. At flag leaf stage, P2 provided the greatest decrease in MDA content. Compared with other seed priming agents, P3 showed the moderate ability to affect MDA content of V1. Lower MDA content of V2 was recorded at P1 at elongation stage (0.020 μmol g^-1^) and at P1 at flag leaf stage (0.019 μmol g^-1^). However, at the last two growth stages, the smallest MDA content was observed at P2 (0.014 and 0.014 μmol g^-1^ for V1 and V2, respectively). Among all the treatments, V2P2 had the lowest MDA content at heading stage (0.014 μmol g^-1^).

**Table 4:** Effects of seed priming on MDA of sorghum under salt stress at different growth stages.

Variety	Seed priming	MDA (μmol g^-1^ FW)			
		Elongation	Flag leaf	Booting	Heading
**V1**	**C**	0.057 a	0.031 a	0.029 a	0.026 ab
	**P1**	0.015 h	0.019 bc	0.014 d	0.019 bc
	**P2**	0.018 gh	0.016 cde	0.019 bc	0.019 bc
	**P3**	0.025 ef	0.017 cde	0.018 bcd	0.020 bc
	**P4**	0.028 de	0.018 bcd	0.020 b	0.024 b
**V2**	**C**	0.055 a	0.021 b	0.027 a	0.033 a
	**P1**	0.041 b	0.013 e	0.016 bcd	0.022 b
	**P2**	0.034 c	0.019 bc	0.014 cd	0.014 c
	**P3**	0.020 fg	0.015 de	0.018 bcd	0.020 bc
	**P4**	0.032 cd	0.018 bcd	0.016 bcd	0.020 bc
**Variety**		**	**	ns	ns
**Seed priming**		**	**	**	**
**Variety×Seed priming**		**	**	ns	ns

## Discussions

Abiotic stresses, such as salinity, heavy metals, ultraviolet radiation, insufficient or excessive water, and low or high temperature, are harmful for the growth and development of plants [23]. Salinity reduces not only leaf size and number but also the growth of tiller and stem as well as plant dry weight [24]. It is essential to improve crop salt tolerance to make use of saline lands. Seed priming is one of the methods to combat the adverse effects of abiotic stresses [25–27]. In this study, we investigated the alleviated effects of four seed priming agents on two salt-stressed sorghum varieties. It is found that seed priming improved salt-stressed sorghum growth by enhancing antioxidative defense. This research provided a reference for the selection of salt-tolerant crop and for the application of seed priming in saline soils.

Aymen et al. reported that agronomic traits and crop yield under salt stress were effectively enhanced by seed priming [28]. In this study, seed priming increased sorghum fresh weight and dry weight. As studied by Khan et al., seed priming uplifted biological yield (g plant^-1^) [29]. For V1, P3 enhanced fresh weight and dry weight to the highest level, while P2 and P3 showed the better promoting effects on fresh weight and dry weight of V2. These results are consistent with Naz et al., who reported that KCl-primed seeds showed significantly higher fresh and dry biomass of seedlings at high salt levels [16]. Many crops at germination and seedling emergence stages are more sensitive to adverse growth conditions, and high-quality germination and emergence can remarkably contribute to uniform crop stand and establishment, leading to higher yield [30]. Our results showed that the biomass of V2 was higher than that of V1. The significant difference in biomass attributes not only to genetic makeup but also to growing environment [31]. Besides, compared to V1, it is clear that V2 had higher water content.

Under saline environment, the accumulation of MDA as a lipid peroxidation product in plant tissues indicates that salt induces oxidative damage, which results in injury in membranes [32]. In this study, seed priming decreased MDA content, which ameliorated salt-mediated membrane injury and consequently alleviated the damaging influences of high NaCl salinity on plant growth. Of the four priming agents, P2 had the best effects in decreasing MDA content of two sorghum cultivars. A similar result was reported by Ellouzi et al., who found that MDA content was reduced by salt stress in the roots and leaves of seedlings from primed seeds in contrast to the salt-stressed seedlings from unprimed seeds [33]. Seed priming usually produce profound effects on seeds. These effects are deposited in the primed seeds and can be recalled when a following stress is imposed. Under this situation, the primed seeds are ready to respond positively to this stress [34]. We also noticed that MDA content of V1 was higher than that of V2, suggesting V2 was higher salt tolerant than V1.

Osmotic regulation is considered a key factor of salt stress tolerance, including the accumulation of various osmotic substances such as sugar, proline and glycine betaine [35]. Proline is a multi-functional molecule that has vital functions of scavenging free radicals, regulating osmotic potential, maintaining membrane integrity, and responding adaptively to salt stress by increasing its accumulation and concentration in plant cells [36]. In the current research, sorghum treated with seed priming accumulated more proline content, especially in the V1P4 and V2P3 treatments. The increase in proline content can stimulate the antioxidant defense mechanism by osmotic regulation and the protection of integrity of cell membranes, thereby decreasing the adverse effects of ROS [37, 38]. Our study found that V1 had higher proline content compared with V2. This may be due to different genetic makeup of the two varieties.

Salinity stress generally causes the accumulation of ROS, which has a vital impact on plant signal transduction, metabolism, photosynthesis, and other physiological and biochemical processes [39]. The antioxidative system, composed of non-enzymatic and enzymatic components, plays a significant role in neutralizing the harmful impacts of reactive oxygen species (ROS) and free radicals [12]. Our research depicted that sorghum seeds with seed priming showed higher levels of CAT, POD and SOD activity. These findings further verified that seed priming alleviated the direct effects of salinity stress. These results are consistent with Jiang et al., who reported that seed priming improved the activities of antioxidant enzymes (SOD, POD, and CAT) in seedlings under salt stress, and consequently reduced the oxidative damage caused by salt [40]. It seems that the beneficial impacts of seed priming agents are different between both varieties investigated. For V1, P2 had the best effects on increasing CAT and POD activity, but the effects of various seed priming agents on CAT and POD activity varied in V2. In agreement with our results, Jisha and Puthur reported that the availability of priming strategy is greatly dependent on seed physiology, plant species, and the nature of priming agent [41]. Interestingly, in our study, we noticed that seed pretreatment with MgSO_4_ (P4) was more beneficial for increasing SOD activity of both V1 and V2 when compared with other treatments. Kanjevac et al. pointed out that priming with MgSO_4_ was beneficial to increase leaf water content, photosynthetic pigments concentration, and protein content in oat [42]. In our study, V2 had higher CAT, POD and SOD activity as compared with V1. Considering this as well as the lower MDA content, it may be the reason that V2 achieved higher biomass amount as compared with V1.

## Conclusion

Seed priming and variety had significant effects on sorghum growth and biochemical parameters. In the present research, seed priming increased sorghum fresh and dry weight, proline content and CAT, POD and SOD activity, and decreased MDA content. In view of the roles of these compounds in the response of plants to environmental stresses, seed priming in present study seemed to have mitigated the negative effects of salt stress on sorghum. Among the four seed priming agents, NaCl and KCl were the optimum priming agents to moderate the adverse effects of salinity stress. Compared to CFSH30 (V1), sorghum ‘1230’ (V2) had higher salt tolerance and greater potential to be planted in saline soils.

## Supporting information

S1 Data The data of this manuscript. (XLSX)
